# A207 SLING-FIBER PRESERVATION DURING POEM IS AN INDEPENDENT FACTOR IN REDUCING POST-POEM GERD SYMPTOMS: A CANADIAN SINGLE-CENTER RETROSPECTIVE STUDY

**DOI:** 10.1093/jcag/gwad061.207

**Published:** 2024-02-14

**Authors:** Y Fujiyoshi, M Fujiyoshi, K Khalaf, G May, C Teshima

**Affiliations:** Division of Gastroenterology, St. Michael's Hospital, University of Toronto, Toronto, ON, Canada; Division of Gastroenterology, St. Michael's Hospital, University of Toronto, Toronto, ON, Canada; Division of Gastroenterology, St. Michael's Hospital, University of Toronto, Toronto, ON, Canada; Division of Gastroenterology, St. Michael's Hospital, University of Toronto, Toronto, ON, Canada; Division of Gastroenterology, St. Michael's Hospital, University of Toronto, Toronto, ON, Canada

## Abstract

**Background:**

Gastroesophageal reflux disease (GERD) after peroral endoscopic myotomy (POEM) has been a limiting factor with POEM procedure. Preservation of the sling-fiber during POEM was reported to reduce post-POEM GERD in Japan, but there are no reports of this technique in a western population.

**Aims:**

The aim is to investigate the association of sling-fiber preservation during POEM and post-POEM GERD symptoms at our institution, which is a large therapeutic endoscopy referral center in Canada.

**Methods:**

This is a retrospective, single-center study of patients who underwent POEM from October 2017 to January 2023 at our center. The initial cohort of patients were treated by conventional POEM until June 2021, after which a second cohort underwent POEM with sling-fiber preservation, as the techniques advanced. The primary outcome was the rate of positive GERD symptoms after POEM. The secondary outcomes were clinical success rate, adverse events rate, use of PPI at follow-up. Multivariate regression was performed to identify factors that reduce post-POEM GERD symptoms.

**Results:**

148 POEM cases (52.5±15.6y/o, female:61(43%)) were included in this study. There was no significant difference in patient characteristics between the groups. The mean procedure time (108.6±34.5 vs 109.1±45.7 min, P=0.93) and rate of adverse events (20% vs 18%, P=0.85) were similar between the traditional and modified groups. In the sling fiber preservation group, gastric myotomy length was significantly longer (2.2±0.7 vs 1.6±0.8 cm, Pampersand:003C0.05) yet the GERD symptom rate at follow-up was significantly lower (22% vs 42%, Pampersand:003C0.05), although PPI use was similar (52% vs 48%, P=0.73). The clinical success rate was similar between groups (88% vs 84%, P=0.6). Regression analysis indicated that, after adjusting for other risk factors of post-POEM GERD, sling-fiber preservation during POEM had an odds ratio of 0.24 (95%CI:0.07-0.85, Pampersand:003C0.05) for post-POEM GERD symptoms.

**Conclusions:**

Sling-fiber preservation during POEM effectively reduces post-POEM GERD symptoms, despite a longer gastric myotomy length. It stands as a independent factor in reducing post-POEM GERD symptoms. Hence, sling-fiber preservation may be a beneficial solution for reducing post-POEM GERD symptoms in Western populations.

Outcomes of conventional POEM and sling-fiber preservation POEM

POEM; Peroral endoscopic myotomy, SD; standard deviation, GERD; gastroesophageal reflux disease, PPI; proton pump inhibitor

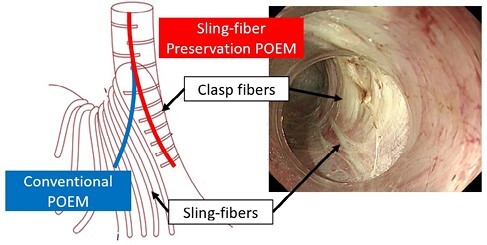

**Funding Agencies:**

None

**Table:** 

	Conventional POEM (N=74)	Sling-fiber preservation POEM (N=74)	P value
Procedure time, mean (SD), min	109.1 (45.7)	108.6 (34.5)	0.93
Gastric myotomy length, mean (SD), cm	1.6 (0.8)	2.2 (0.7)	ampersand:003C0.001
Adverse events (Clavien-Dindo classification≧II)	13 (17.6%)	15 (20.3%)	0.85
Clinical success rate (Eckardt score ≦3) N=138	59 (83.1%)	60 (89.5%)	0.32
GERD symptoms N=138	30 (42.3%)	15 (22.4%)	0.018
PPI usage N=138	34 (47.9%)	35 (52.2%)	0.73

